# Effects of Galectin-1 on Biological Behavior in Cervical Cancer

**DOI:** 10.7150/jca.38538

**Published:** 2020-01-14

**Authors:** Mandika Chetry, Yizuo Song, Chunyu Pan, Ruyi Li, Jianan Zhang, Xueqiong Zhu

**Affiliations:** Department of Obstetrics and Gynecology, the Second Affiliated Hospital of Wenzhou Medical University, Wenzhou, Zhejiang, 325027, China.

**Keywords:** Galectin-1, cervical cancer, proliferation, apoptosis, migration, invasion.

## Abstract

**Background**: We previously revealed that the expression of galectin-1 (LGALS1) was significantly reduced after neoadjuvant chemotherapy treatment in cervical cancer patients. The objective of this study is to investigate the effects of LGALS1 expression on biological behaviors of cervical cancer cells.

**Methods**: Immunohistochemistry and immunocytochemistry were performed to detect the expression of LGALS1 in cervical cancer tissues and cells (SiHa and C33A). Western blot analysis was performed to evaluate the efficacy of lentivirus-mediated upregulation or downregulation of LGALS1 in cervical cancer cells. Cell viability and proliferation were detected by CCK-8 and BrdU assays, respectively. Annexin V-FITC/PI apoptosis detection kit was employed to measure the apoptosis of cervical cancer cells. Transwell invasion and migration assays were also conducted to explore the invasive and migratory capabilities of cervical cancer cells. The expression of apoptosis- (Bcl-2 and Bax), invasion- (MMP-2 and MMP-9), and migration-related (Fascin and Ezrin) proteins, were detected by Western blot analysis. Xenograft mouse model of cervical cancer was generated to explore whether LGALS1 overexpression could promote tumor growth *in vivo*.

**Results**: LGALS1 was overexpressed in cervical cancer tissues and cell lines compared to that in normal cervical tissues and epithelium cells. Upregulation of LGALS1 significantly promoted the cell proliferation, inhibited cell apoptosis, and enhanced the migratory and invasive abilities of both SiHa and C33A cells, whereas downregulation of LGALS1 led to the opposite results. The level of Bcl-2, MMP-2, MMP-9, Fascin, and Erzin expression was significantly upregulated in cervical cancer cells with LGALS1 overexpression, while converse results were obtained in LGALS1 knockdown cancer cells. *In vivo* study also showed that LGALS1 overexpression facilitated tumor growth of cervical cancer cells.

**Conclusion**: Overexpression of LGALS1 significantly promoted and enhanced the aggressive features of cervical cancer both *in vitro* and *in vivo*, which may be associated with high expression of Bcl-2, MMP-2, MMP-9, Fascin, and Erzin proteins.

## Introduction

Cervical cancer, originating from epithelial neoplastic transformation of uterine cervix, is the fourth cause of cancer-related death among women worldwide 1, 2. However, due to inadequate screening protocols for early detection of lesion in cervix, cervical cancer is still considered as the most common malignancy and poses a vital public health problem among Chinese women 3. According to statistics, the number of new cases of cervical cancer in China is estimated to reach 93,500 in 2030 and 186,600 in 2050 4, respectively. Thus, it is required to find the potential diagnostic markers as well as therapeutic targets, aiming to reduce the mortality and improve overall survival in patients with cervical cancer.

Galectin-1 (LGALS1) is a member of the carbohydrate-binding protein family, which shares a conserved amino acid sequence in the carbohydrate recognition domain and shows high affinity for β-galactoside-containing glycol-conjugates 5, 6. Several studies have demonstrated that LGALS1 plays an important role in tumorigenesis, progression and metastasis of human cancers 7, serving as a reliable diagnostic and prognostic biomarker in cancerous patients 8, 9. For instance, Schulkens *et al.* 10 have observed that LGALS1 expression in non-small cell lung cancer tissues was elevated compared with adjacent normal tissues. Additionally, overexpression of LGALS1 has also been reported in hepatocellular carcinoma 11, 12 and bladder urothelial carcinoma 13. Furthermore, upregulation of LGALS1 is also implicated in gastric tumor growth and metastasis 14. However, the role of LGALS1 in cervical cancer is still not fully elucidated. Notably, our previous proteomic study demonstrated that the expression of LGALS1 is significantly reduced after neoadjuvant chemotherapy treatment in cervical cancer patients, which may be associated with neoadjuvant chemotherapy exposure and response 15. Nevertheless, the function of LGALS1 on biological behavior in cervical cancer has not been extensively investigated yet.

In the current study, we explored the expression of LGALS1 protein in cervical cancer tissues and cells compared to adjacent non-tumor tissues and epithelial cells of cervix, respectively. Furthermore, we also investigated the effect of LGALS1 overexpression and knockdown on the proliferation, apoptosis, and migratory and invasive capabilities of cervical cancer cells and the related proteins *in vitro*. More importantly, the influence of LGALS1 on the tumor growth of cervical cancer *in vivo* was further studied.

## Materials and Methods

### Ethics statement

This study was approved by the ethical committee of the Second Affiliated Hospital of Wenzhou Medical University. Informed consent was obtained from each subject for the sample collection and analysis. All animal experiments were carried out according to the Guide for the Care and Use of Laboratory Animals published by the United States National Institutes of Health. They were approved by the Animal Care and Use Committee of Wenzhou Medical University.

### Patients and tissue samples

Women with stage IB-IIA cervical cancers were recruited for this study, who underwent radical hysterectomy at the Second Affiliated Hospital of Wenzhou Medical University between January 2013 and August 2015. All these patients were retrospectively reviewed using electronic medical records. After exclusion of patients without complete clinicopathological data, 20 patients were enrolled in our study with a median age of 43 years (range, 24-59 years). All patients were pathologically diagnosed with squamous cell carcinoma of cervix after surgery (Differentiation: 13 moderate and 7 well; stage: 12 IB and 8 IIA). None of the patients received chemotherapy or radiotherapy prior to surgery. None of the patients had other synchronous malignancies or serious systemic diseases. Formalin-fixed cervical cancer tissues and matching adjacent non-tumor tissues from these patients were used for immunohistochemistry (IHC) staining.

### Cell lines and culture

The human cervical squamous cancer cell lines (SiHa and C33A) and normal cervical epithelial cell (Ect1/E6E7) were purchased from the Type Culture Collection of the Chinese Academy of Sciences (Shanghai, China). All cell lines were cultured in Dulbecco's Modifed Eagle medium (DMEM) (Gibco, USA) supplemented with 10% fetal bovine serum (FBS) (Gibco, USA) and 1% antibiotics (penicillin-streptomycin). All cells were incubated at 37°C in a humidified atmosphere containing 5% CO_2_. Cells were cultured to a confluence of 80% and passaged by using 1× trypsin with 0.2% Ethylene Diamine Tetraacetic Acid (EDTA).

### Immunohistochemistry (IHC) and immunocytochemistry (ICC)

IHC staining was performed using the SPlink Detection Kits (Biotin-Streptavidin HRP Detection Systems, ZSGB-BIO, SP-9000) in accordance with the manufacturer's instruction. Paraffin-embeded sections were cut at 4 μm thickness and deparaffinized in xylene and rehydrated in a gradient of ethanol solutions. After that, the tissue slides were washed with phosphate-buffered saline (PBS), and then placed in 80 mL plastic jars containing citrate buffer (pH 6.0) and repeatedly heated for 20 min at 95°C in a microwave oven for antigen retrieval. Endogenous peroxidase activity was suppressed with 3% hydrogen peroxide in methanol for 15 min and nonspecific binding was prevented through incubation with non-immune serum for 15 min. Tissue sections were then incubated with primary mouse anti-human LGALS1 monoclonal antibody (Santa Cruze, USA, 166618; 1:200) overnight at 4°C, followed by further incubation with biotin-conjugated secondary antibodies for 30 min at room temperature. Subsequently, the samples were exposed to streptavidin peroxidase as a label for 20 min. The sections were stained with diaminobenzidine for 10 min and counterstained with hematoxylin to enhance the nuclear detection. Finally, the slides were mounted, dehydrated through xylene and cover slipped. Appropriate positive and negative controls were stained in parallel. The results were assessed by two independent observers, who were blinded to the study. LGALS1 immunoreactivity was observed in the cytoplasm and cells that showed yellowish brown were recognized as positive. The percentage of positive cells was scored as following: 0 (0-5%), 1 point (6%-24%), 2 points (25%-49%), 3 points (50%-74%), and 4 points (75%-100%). Staining intensity was graded semiquantitatively into four levels as following: 0 (negative), 1 point (weak), 2 points (moderate), and 3 points (strong). The immunoreactive score was derived by the fraction of positive cell scores multiplied by staining intensity score.

Additional ICC analyses of LGALS1 expression were performed in SiHa, C33A and Ect1/E6E7 cells, which were grown on Chamber Slides System (Lab-Tek, USA) in a humidified incubator at 37°C with 5% CO_2_. After 24 h, the cells were fixed with acetic acid and methanol solution (ratio 1:3) at room temperature for 10 min. ICC was conducted using mouse anti-human LGALS1 (overnight incubation at 4°C and 1:200 dilution). The cells were then stained with avidin-biotin-peroxidase complex (UltramarqueTM-HRP-Detection kit, Greenwood, USA). Negative controls were treated identically by using PBS instead of primary antibodies. The ICC staining results were quantified based on the system identical to IHC and evaluated by two individual observers blindly.

### Lentivirus construction and transfection of target cells

The LGALS1 sequences were amplified by polymerase chain reaction and then inserted into the lentiviral expression vector pLVX-IRES-ZsGreen1 (Origene, USA). Four different LGALS1 small hairpin RNAs (shRNAs) and the negative control plasmid were inserted into pGFP-C-shLenti vectors (Origene, USA). After confirmation using gene sequencing, LGALS1-pLVX-IRES-ZsGreen1, LGALS1-pGFP-C-shLenti and the corresponding control vectors, together with two packaging plasmids psPAX2 and pMD2.G (donated by Dr Luzhe Sun, The University of Texas Health Science Center at San Antonio) were transfected into 293T cells for 48 h, using lipofectamine 2,000 (Gibco, USA). Cells were seeded in 60-mm dish and cultured in DMEM medium without antibiotics for 24 h. Then, recombinant lentiviral particles were harvested and filtered to infect cell lines with the help of polybrene (final concentration 8 μg/mL). The expression of LGALS1 in all transfected cells were confirmed by Western blot. The primers and shRNA used for gene upregulation or downregulation were listed in Table 1.

### Western blot analysis

The cells were washed with PBS and then lysed in a buffer (40 mM Tris-Cl, 10 mM EDTA, 120 mM NaCl, 0.1% Nonidet P-40) containing protease inhibitor cocktail (Beyotime, China). The lysates were incubated on ice for 30 min, followed by centrifugation at 12,000 *g* at 4°C for 20 min. The protein concentration was determined by a Bicinchoninic Acid Protein Assay Kit (Beyotime, China). Equal amounts of protein (30 μg) were separated by 12% sodium dodecyl sulfate-polyacrylamide gel (SDS-PAGE) and then transferred onto a polyvinylidene difluoride (PVDF) membrane (Millipore, USA). The membranes were blocked for 2 h with 5% skimmed milk in Tris-buffered saline with 0.1% Tween-20 (TBS-T, pH 7.4) at room temperature and then incubated overnight at 4°C with one of the appropriate primary antibodies at 1:2000 dilutions, unless specified otherwise. The primary antibodies used in this study were as following: mouse anti-human LGALS1 antibody (Santa Cruze, USA, 166618), rabbit anti-human Bcl-2 antibody (Abcam, USA, ab59348), rabbit anti-human Bax antibody (Abcam, USA, ab199677), mouse anti-human MMP-2 antibody (Abcam, USA, ab2462), rabbit anti-human MMP-9 antibody (Abcam, USA, ab38898), rabbit anti-human Fascin antibody (Abcam, USA, ab126772), rabbit anti-human Erzin antibody (Abcam, USA, ab47293), mouse anti-human α-Tubulin antibody (Abcam, USA, ab7291), and rabbit anti-human GAPDH antibody (Abcam, USA, ab181602). The membranes were rinsed and incubated with a goat anti-rabbit (Abcam, USA, ab205718) or anti-mouse (Abcam, USA, ab205719) horseradish peroxidase-conjugated secondary antibody at a concentration of 1:4000 for 2 h at room temperature. Finally, immunoreactive bands were visualized with an enhanced chemiluminescence detection reagent (Beyotime, China). The films were scanned using a GS-800 scanner and Quantity One software (Bio-Rad, USA) was employed to analyze the visualized protein bands. The expression levels of above proteins were quantified with densitometry and normalized by corresponding levels of α-Tubulin or GAPDH, respectively. All experiments were performed in triplicate.

### Cell viability assay

Viability of cervical cancer cells was assessed using Cell Counting Kit-8 (CCK-8) assay (Dojindo, Japan). Briefly, cells (2000 cells/well) from the control, overexpression and knockdown groups were dispensed into 96-well plates with a volume of 100 μl/well and incubated at 37°C in 5% CO_2_ for 48 h. Subsequently, 10 μl CCK-8 solution in 100 μl complete DMEM was added to each well, followed by incubation for 4 h. After that, the absorbance of each well at 450 nm was detected using Micro-plate Reader (Bio-Tek Instruments, USA). Cell viability (%) was calculated by average absorbance of transfected group/average absorbance of control group × 100%. The experiment was repeated three times.

### Cell proliferation assay

BrdU incorporation assay (Calbiochem, USA) was employed to determine cell proliferation. Briefly, transfected or non-transfected cells were seeded into 6-well plate with a density of 1 × 10^5^ cells/well for 48 h. Then, the cells were incubated with BrdU (1 mg/ml) for 4 h and stained with anti-BrdU antibody (Cell Signaling Technology, USA) according to the manufacturer's instruction. After that, the number of BrdU positive (+) cells in each well was counted under microscope (Nikon, Japan), which was proportional to cell proliferation.

### Cell apoptosis assay

Apoptosis was analyzed using Annexin V-FITC/PI apoptosis detection kit (BD Pharmingen, USA) according to the manufacturer's instructions. Briefly, the cells in 80% confluency at 48 h were collected with 0.25% trypsin, washed with pre-cooled PBS for twice, and stained using kit solution for 15 min at room temperature in the dark. The rate of apoptotic cells was recorded using flow cytometry (Beckman Coulter, USA). Data were quantified by FlowJo sofeware (Tree Star Incorporation, USA).

### Transwell migration and invasion assays

The abilities of cell migration and invasion were assessed in 24-well plates with 8 μm pore transwell polycarbonate filters (Corning, USA). For the migration assay, cells with or without transfection were resuspended in serum-free DMEM and seeded to the upper chambers without Matrigel pre-coating at 1 × 10^4^ cells/well, while DMEM containing 2.5% FBS was added to the lower chamber. Following incubation at 37°C in 5% CO_2_ for 48 h, the filters were fixed in 4% paraformaldehyde at room temperature for 20 min and stained with 0.1% crystal violet (Beyotime, China) for 10 min. Then, cells on the upper surface of the filter were removed using a cotton swab gently. Images of the cells migrated to the lower side of the filter were captured (magnification, × 40) and counted (magnification, × 100) under microscope (Nikon, Japan) by an independent investigator.

For the invasion assay, the upper transwell inserts were pre-coated with 100 μl 1:5 diluted Matrigel (BD Biosciences, USA), where cells from each group were seeded next. The medium in the lower chamber was complete DMEM with 10% FBS. After incubation at 37°C in 5% CO_2_ for 48 h, a cotton swab was used to gently remove the cells and Matrigel on the upper surface of the membrane. The number of invaded cells was determined in the same way as stated for the migration assay. All experiments were repeated in triplicate.

### Animal studies

Four-week-old female nude mice were purchased from Beijing Vital River Laboratory Animal Technology of China, which were housed under specific pathogen free (SPF) condition in Wenzhou Medical University and used for *in vivo* animal experiments. Exponentially growing SiHa and C33A cells with LGALS1 overexpression (LGALS1-Lt), LGALS1 knockdown (LGALS1-shRNA), or corresponding empty vectors (Control-Lt and Control-shRNA) were suspended in 50% Matrigel in cold PBS and injected subcutaneously into the right flank of the mice (4 × 10^5^ cells/mouse, 7 mice/group). One week after inoculation, the length (a) and width (b) of tumor tissue were monitored using a caliper once a week. The tumor volumes were calculated by the following formula: volume (mm^3^) = a*b^2^/2. Mice were sacrificed after tumor inoculation for a total of six weeks. Then the tumor samples were dissected, weighed and imaged with the camera.

### Statistical analyses

All data analyses were performed using SPSS software (version 18.0; Chicago, USA). Continuous variables were expressed as the means ± SD. Two-tailed Student *t* test was applied for comparison of continuous variables. When group numbers were more than two, One-way ANOVA analysis was performed to determine the difference. *P* <0.05 was defined as statistical significance.

## Results

### LGALS1 is overexpressed in cervical cancer tissues and cell lines

**Figure 1:**
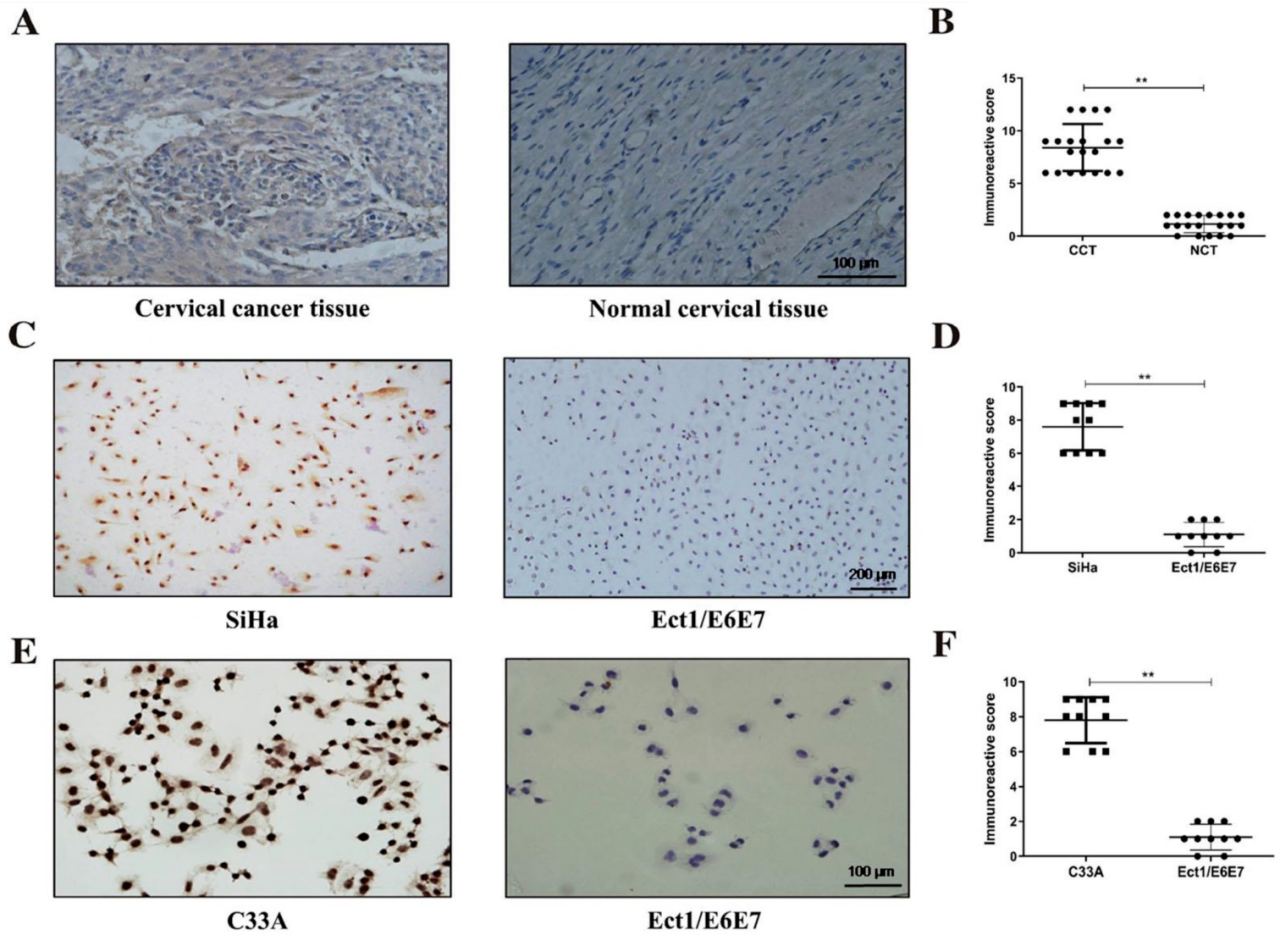
LGALS1 is overexpressed in cervical cancer tissues and cells. (A and B) Representative images and scores of IHC for LGALS1 in human cervical cancer tissue and matched normal cervical tissue (×200). (C, D, E and F) Representative images and scores of ICC for LGALS1 in SiHa cells, C33A cells and Ect1/E6E7 cells (×100). Values are expressed as the mean ± SD. *P <0.05, **P <0.01. LGALS1, galectin-1; IHC, immunohistochemistry; ICC, immunocytochemistry; CCT, cervical cancer tissue; NCT, normal cervical tissue.

To understand the expression of LGALS1 in cervical cancer, we conducted IHC analyses of 20 cervical cancer specimens and matched non-tumor tissues in this study. Significantly higher expression of LGALS1 was exhibited in cervical cancer tissues (mainly in the cytoplasm) than normal cervical samples, while the latter showed only weak expression of LGALS1 (Figure 1A). The mean IHC scores of LGALS1 in cervical cancer and normal cervical tissues were 8.40 ± 2.23 and 1.15 ± 0.81, respectively. A significant difference in LGALS1 expression was observed between cervical cancer and adjacent non-tumor cervical tissues (*P* <0.01; Figure 1B).

To further validate the results of IHC, additional ICC analyses of LGALS1 expression were also performed in SiHa and C33A cervical cancer cells compared to normal cervical Ect1/E6E7 cells. As shown in Figure 1C and 1E, LGALS1 protein expression in the SiHa and C33A cells was detected mainly in the cytoplasm and nucleus, whereas the Ect1/E6E7 cells reflected nearly negative staining of LGALS1. The immunoreactive score of ICC for LGALS1 expression was significantly increased in the SiHa (7.60 ± 1.43) and C33A (7.80 ± 1.32) cell lines compared with Ect1/E6E7 cells (1.10 ± 0.74) (*P* <0.01; Figures 1D-1F).

### Lentivirus-mediated upregulation and downregulation of LGALS1 in SiHa and C33A cells

**Figure 2:**
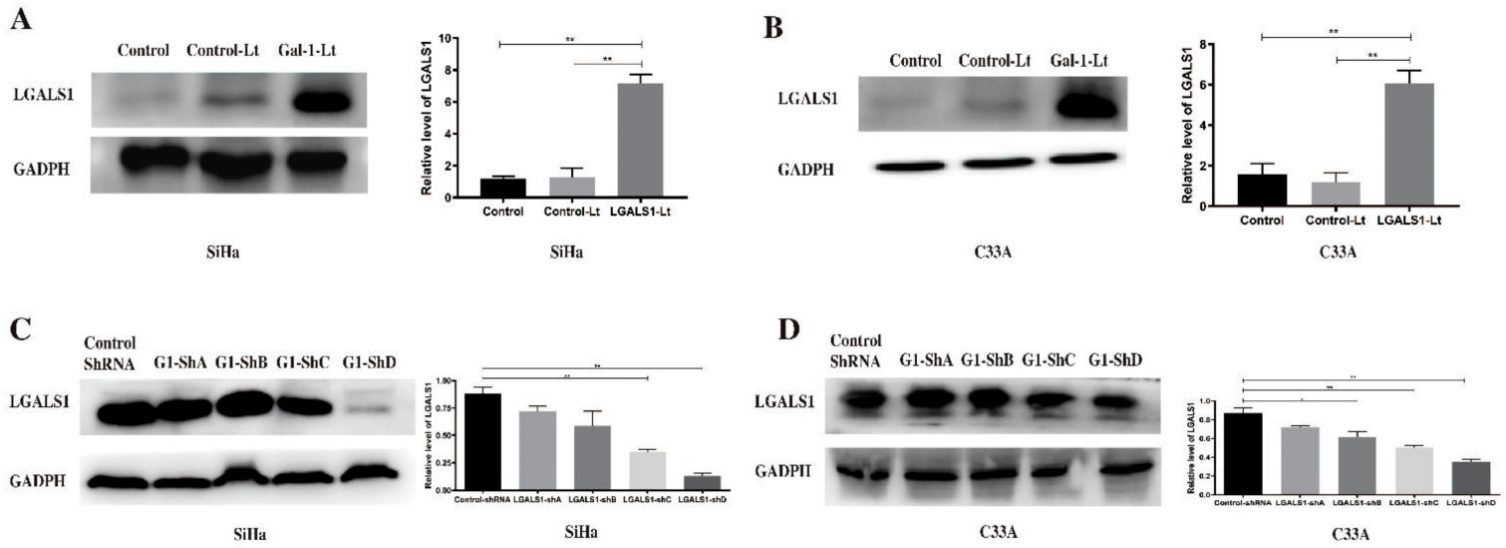
LGALS1 is upregulated or downregulated by LGALS1-Lt or LGALS1-shRNA in both SiHa and C33A cell lines. (A and B) Protein expression of LGALS1 in SiHa and C33A cells transfected with LGALS1-Lt was determined by Western blot analysis at 48 h post-transfection. (C and D) LGALS1 protein expression in SiHa and C33A cells transfected with four various LGALS1 shRNAs was detected by Western blot analysis 48 h post-transfection. Values are expressed as the mean ± SD. *P <0.05, **P <0.01. LGALS1, galectin-1; Control-Lt, control lentivirus; LGALS1-Lt, galectin-1 lentivirus; shRNA, small hairpin RNA.

As shown in Figure 2, the efficacy of upregulation and downregulation of LGALS1 mediated by lentivirus was confirmed using Western blot analysis. Transfection of both SiHa and C33A cells with LGALS1-pLVX-IRES-ZsGreen1 lentivirus markedly increased the expression of LGALS1 by approximately 4-fold (*P* <0.01; Figures 2A-2B). In comparison, approximately 20%-85% reduction of LGALS1 expression was shown in both SiHa and C33A cells transfected with LGALS1-shRNA (*P* <0.01; Figures 2C-2D). The levels of LGALS1 expression in empty-vector group showed no significant difference compared to the blank control group (*P* >0.05). Therefore, LGALS1-shRNA-D was used in the following experiments.

### Overexpression of LGALS1 enhances the viability and promotes the proliferation of SiHa and C33A cells

**Figure 3:**
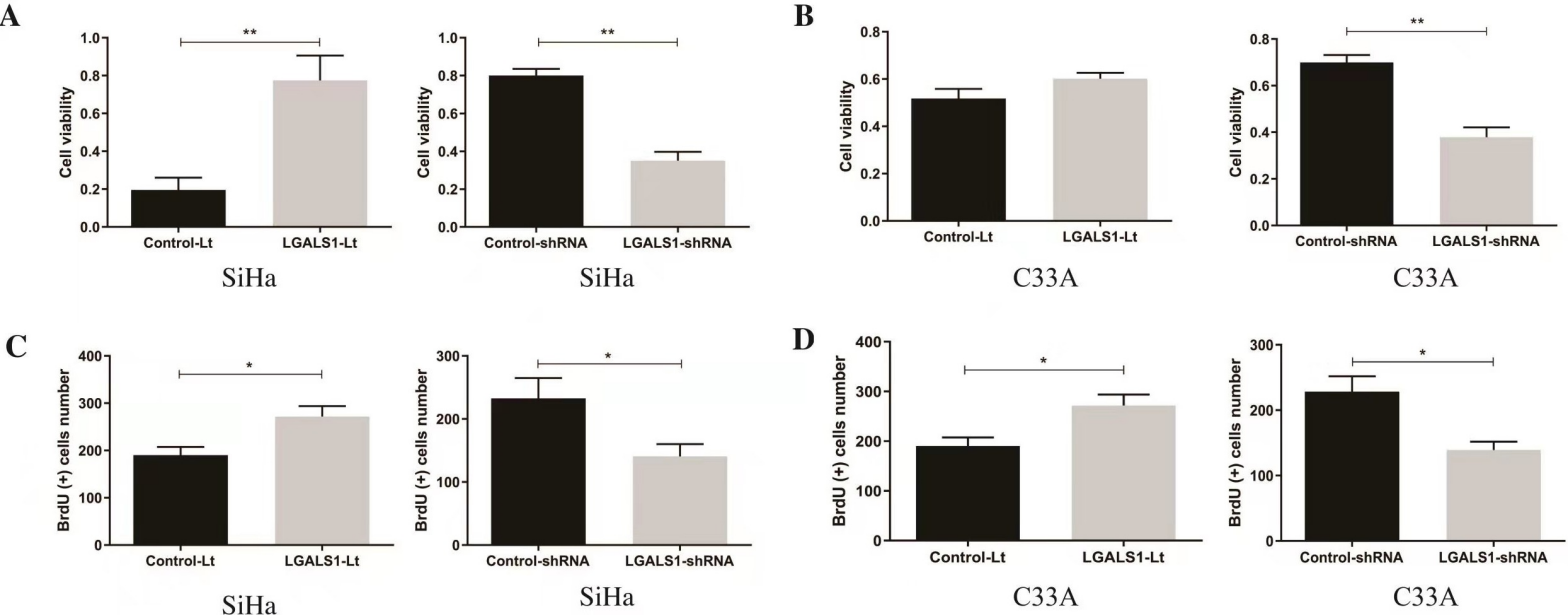
LGALS1 promotes proliferation in both SiHa and C33A cell lines. (A and B) The viability of SiHa and C33A cells at 48 h after transfection with LGALS1-Lt or LGALS1-shRNA were measured using CCK-8 assay. (C and D) The proliferation of SiHa cells at 48 h after transfection with LGALS1-Lt or LGALS1-shRNA-D were assessed using BrdU assay. Values are expressed as the mean ± SD. *P <0.05, **P <0.01. LGALS1, galectin-1; LGALS1-Lt, galectin-1-lentivirus; shRNA, small hairpin RNA; CCK-8, cell counting Kit-8.

At 48 h after transfection, the effects of LGALS1 on the viability and proliferation of SiHa and C33A cells were assessed using CCK-8 and BrdU assay, respectively. A significant decrease of cell viability and proliferation were detected in the LGALS1-shRNA group compared with that in the control-shRNA group (*P* <0.01, Figures 3A-3B; *P* <0.05, Figures 3C-3D). While transfection with LGALS1-Lt did not dramatically influence the viability of C33A cells (*P* >0.05; Figure 3B), the viability was remarkably increased in SiHa cells following transfection (*P* <0.01, Figure 3A). Moreover, BrdU positive rates were significantly enhanced in both SiHa and C33A cells with LGALS1 overexpression (*P* <0.05, Figures 3C-3D).

### Overexpression of LGALS1 inhibits the apoptosis of SiHa and C33A cells

**Figure 4:**
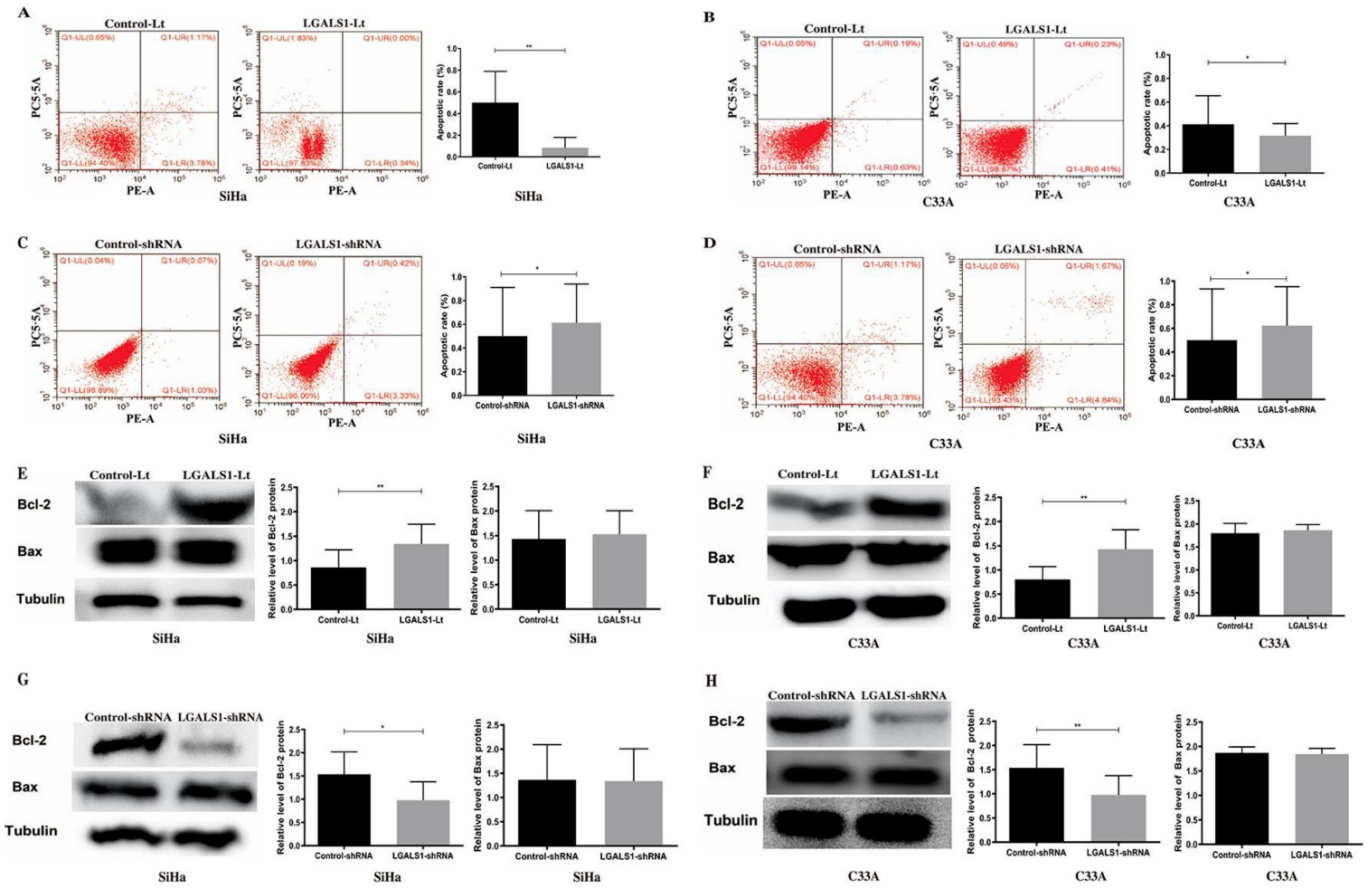
LGALS1 inhibits apoptosis in both SiHa and C33A cell lines. (A, B, C and D) The apoptosis of SiHa and C33A cells with LGALS1 overexpression or knockdown were analyzed by flow cytometric analysis. (E, F, G and H) Levels of apoptosis-related proteins Bcl-2 and Bax in SiHa and C33A cells with LGALS1 overexpression or knockdown were detected by Western blot analysis. Values are expressed as the mean ± SD. *P <0.05, **P <0.01. LGALS1, galectin-1; LGALS1-Lt, galectin-1-lentivirus; shRNA, small hairpin RNA.

The role of LGALS1 on cell apoptosis was further studied and flow cytometry was utilized to measure the apoptotic cells. As shown in Figure 4, SiHa and C33A cells with LGALS1 overexpression had significantly lower apoptotic rate (*P* <0.01, Figure 4A; *P* <0.05, Figure 4B) whereas LGALS1 knockdown weakly enhanced the apoptotic rate in both SiHa and C33A cells (*P* <0.05; Figures 4C-4D). Moreover, the level of apoptosis-related protein Bcl-2 was significantly elevated in both SiHa and C33A cells after upregulation of LGALS1 (*P* <0.01; Figures 4E-4F), and reduced after LGALS1 knockdown (*P* <0.05, Figure 4G; *P* <0.01, Figure 4H). However, the expression level of Bax protein was not changed in SiHa and C33A cells after LGALS1 overexpression or knockdown (*P* >0.05; Figures 4E-4H).

### Overexpression of LGALS1 promotes the migratory and invasive capabilities of SiHa and C33A cells

**Figure 5:**
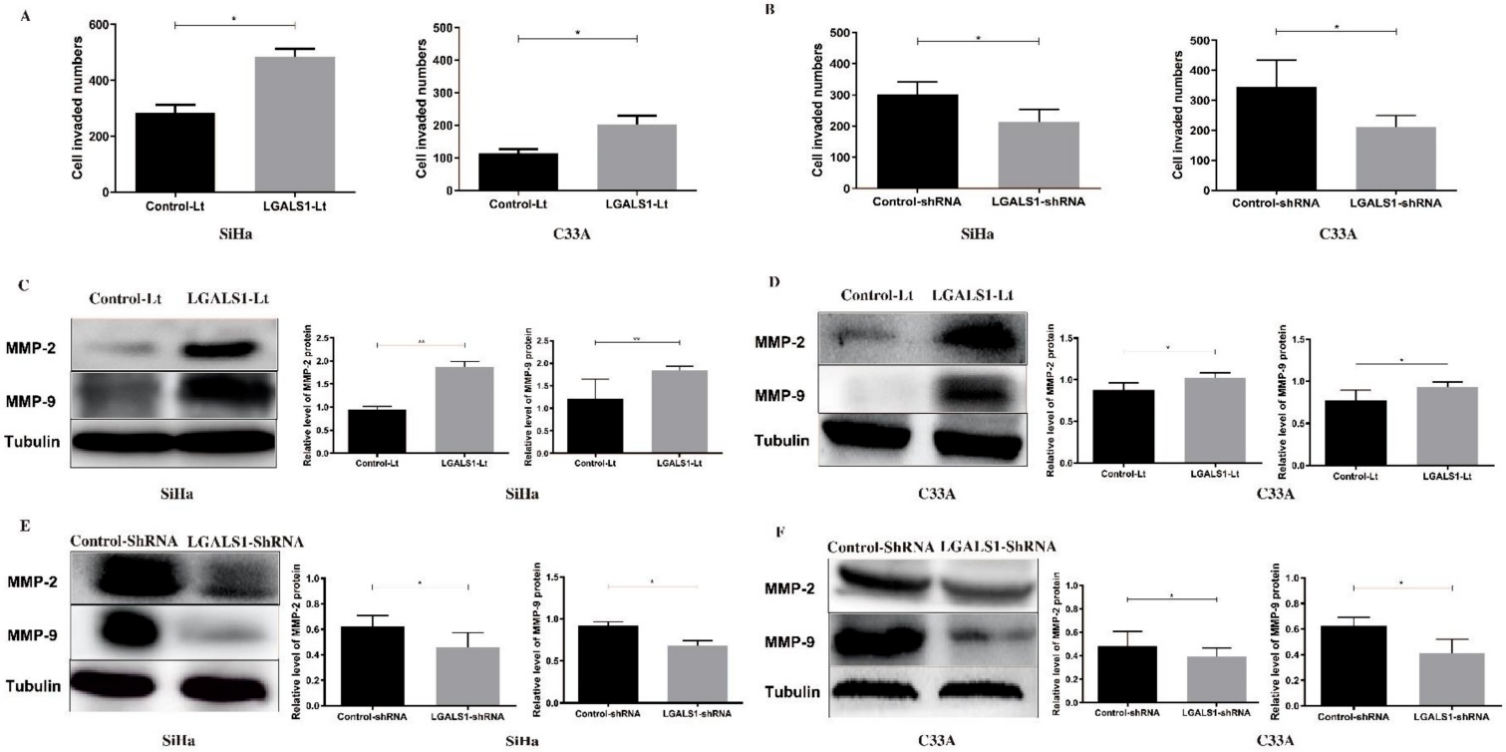
LGALS1 enhances invasive abilities in both SiHa and C33A cell lines. (A and B) Quantification of invaded SiHa and C33A cells with LGALS1 overexpression or knockdown were counted under microscope. (C, D, E and F) Levels of invasion-related proteins MMP-2 and MMP-9 in LGALS1 overexpression or knockdown SiHa and C33A cells were detected by Western blot analysis. Values are expressed as the mean ± SD. *P <0.05, **P <0.01. LGALS1, galectin-1; LGALS1-Lt, galectin-1-lentivirus; shRNA, small hairpin RNA; MMP, matrix metalloproteinases.

**Figure 6:**
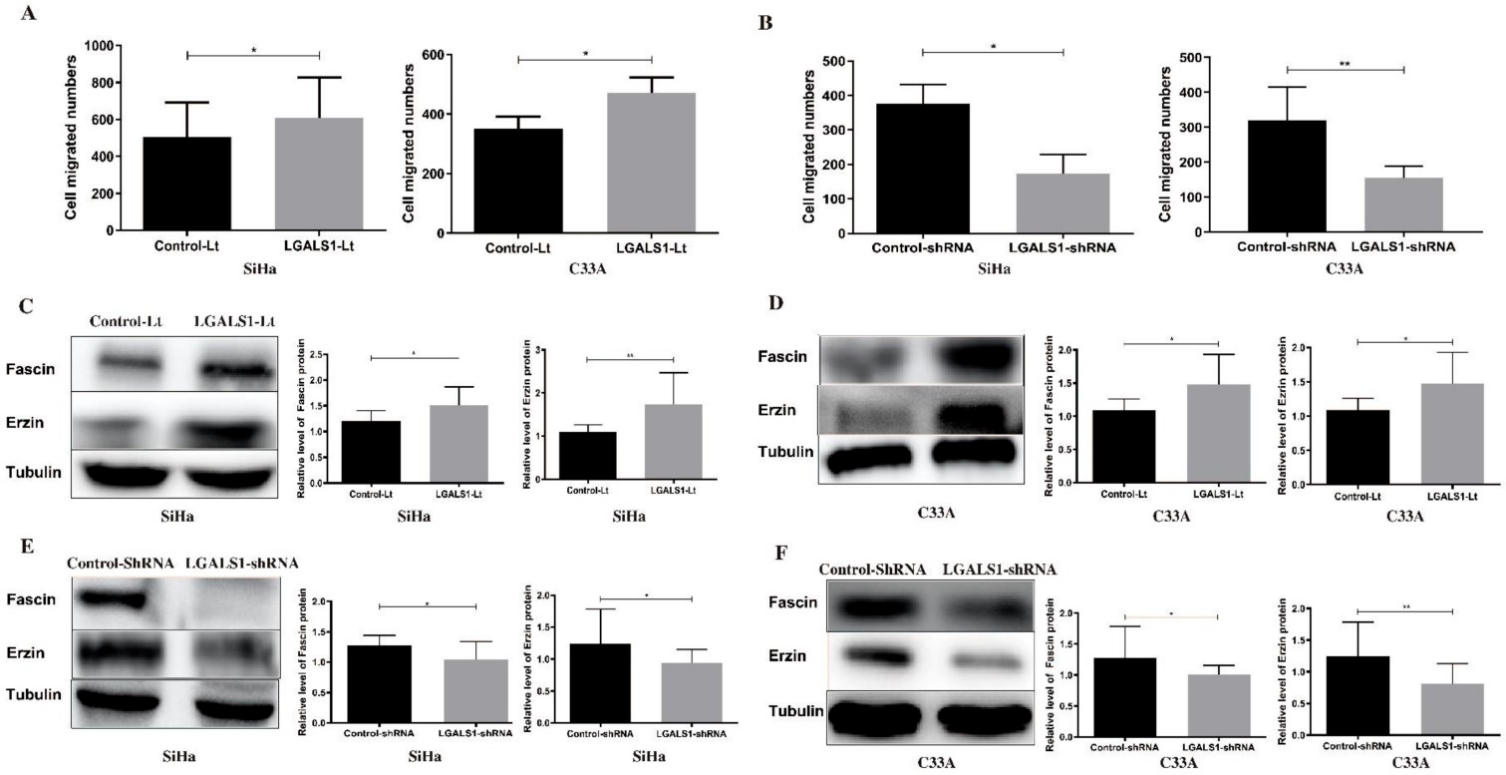
LGALS1 enhances migration abilities in both SiHa and C33A cell lines. (A and B) Quantification of migrated SiHa and C33A cells with LGALS1 overexpression or knockdown were counted under microscope. (C, D, E and F) Levels of migration-related proteins Fascin and Erzin in LGALS1 overexpression or knockdown SiHa and C33A cells were detected by Western blot analysis. Values are expressed as the mean ± SD. *P <0.05, **P <0.01. LGALS1, galectin-1; LGALS1-Lt, galectin-1-lentivirus; shRNA, small hairpin RNA.

Subsequently, the effect of LGALS1 on the invasive (Figure 5) and migratory (Figure 6) capabilities of SiHa and C33A cell lines was examined using transwell assay. For both SiHa and C33A cells, a significant increase of cell invasion (*P* <0.05; Figure 5A) and migration (*P* <0.05; Figure 6A) were detected in the LGALS1-Lt group compared with that in the control-Lt group. Conversely, LGALS1 knockdown markedly decreased the invasive (*P* <0.05; Figure 5B) and migratory (*P* <0.05 for SiHa, *P* <0.01 for C33A; Figure 6B) abilities of both SiHa and C33A cells compared with control-shRNA cells.

Next, the expression of invasion and migration related proteins were evaluated by Western blot analyses, including MMP-2, MMP-9, Fascin and Erzin. The expression of MMP-2, MMP-9, Fascin, Erzin were dramatically increased in LGALS1 overexpressing SiHa and C33A cells compared to the control-Lt cells (*P* <0.01, Figures 5C; *P* <0.01, Figure 5D; *P* <0.05 for Fascin, *P* <0.01 for Erzin, Figure 6C; *P* <0.05, Figure 6D). On the contrary, downregulation of LGALS1 significantly suppressed the expression of MMP-2, MMP-9, Fascin and Erzin protein expressions in both SiHa and C33A cells (*P* <0.05, Figure 5E; *P* <0.05 for MMP-2, *P* <0.01 for MMP-9, Figure 5F; *P* <0.05, Figure 6E; *P* <0.05 for MMP-2, *P* <0.01 for MMP-9, Figure 6F).

### Overexpression of LGALS1 promotes tumor growth of cervical cancer in vivo

**Figure 7:**
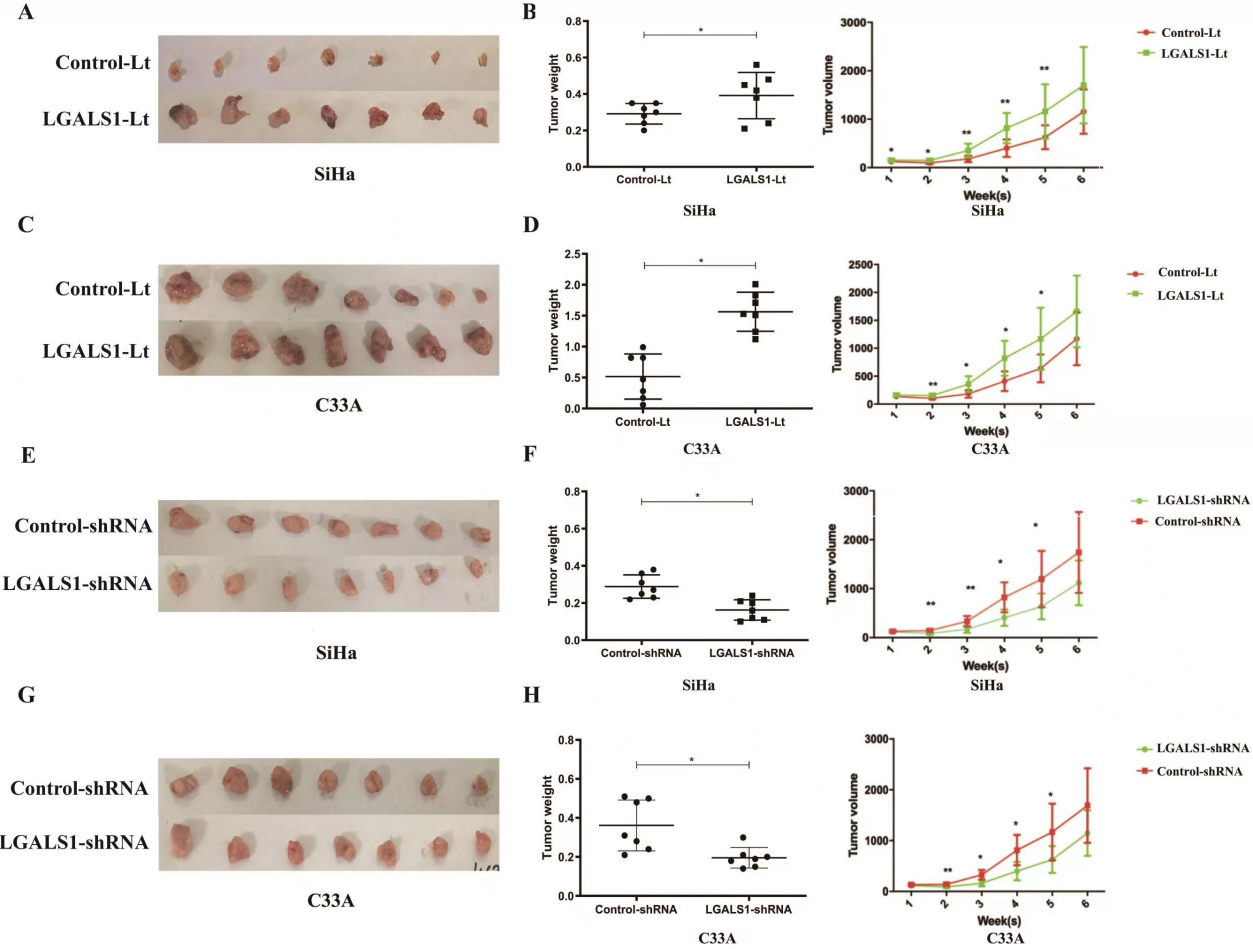
LGALS1 promotes cervical cancer growth *in vivo*. (A and C) Overexpression of LGALS1 promoted SiHa and C33A xenografts growth. (B and D) Tumor volumes and weights were measured in LGALS1 overexpression SiHa and C33A xenografts nude mice. (E and G) Knockdown of LGALS1 inhibited SiHa and C33A xenografts growth. (F and H) Tumor volumes and weights were measured in LGALS1 knockdown SiHa and C33A xenografts nude mice. Values are expressed as the mean ± SD. *P <0.05, **P <0.01. LGALS1, galectin-1; LGALS1-Lt, galectin-1-lentivirus; shRNA, small hairpin RNA.

To further investigate whether LGALS1 promotes tumor growth *in vivo*, subcutaneous cervical cancer xenografts in nude mice were generated by injection with SiHa and C33A cells transfected with LGALS1-Lt, LGALS1-shRNA and related empty control vectors respectively. The diameter of the implanted tumors was measured every week. Six weeks after subcutaneous implantation (both for SiHa and C33A), tumors in the LGALS1-Lt group were larger and heavier than that in the control-Lt group (*P* <0.05; Figures 7A-7D), while tumors in LGALS1-shRNA group were remarkably smaller and lighter than that in the control-shRNA group (*P* <0.05; Figure 7E-7H). Tumor volumes (both for SiHa and C33A) were significantly higher in the LGALS1-Lt group than that in the control-Lt group from week 2 onwards (*P* <0.01; Figures 7B and 7D), while apparently smaller in the LGALS1-shRNA group compared with the control-shRNA group from the 2nd weeks after inoculation (*P* <0.05; Figures 7F and 7H).

## Discussion

In this study, we observed that LGALS1 was upregulated in cervical cancer tissues and cell lines, and it was significantly associated with aggressive behavior of cervical squamous carcinoma. One study has shown that LGALS1 expression was increased remarkably according to the pathologic grade of cervical tissues 16. In our study, overexpression of LGALS1 was also observed in human cervical cancer tissues as compared with matched non-tumor samples using IHC staining, corresponding with previous reports 17-19. Furthermore, similar results were obtained in ICC studies that level of LGALS1 expression was significantly elevated in SiHa and C33A cell lines comparing to normal epithelial cells of cervix. Therefore, these findings together with previous reports strongly suggest that LGALS1 is overexpressed in cervical cancer cells 20, 21.

To further investigate the effects of LGALS1 on multiple biological functions of human cervical cancer cells, we employed lentivirus-mediated vectors to successfully overexpress and knockdown endogenous LGALS1 in SiHa and C33A cell lines. Cell viability is defined as the amount of living cells in a certain population and apoptosis of cells is an important indicator for action of cell survival or death when exposing to various stimulations 22. Recently, Kim *et al.* 23 has reported that LGALS1 promoted the proliferation of human keratinocytes and fibroblasts thereby accelerating wound healing, which may be achieved via triggering the accumulation of intracellular reactive oxygen species (ROS) 24. Similar to neuroblastoma 25, oral squamous cell carcinoma, lung cancer 26, and breast cancer 27, downregulation of LGALS1 had been shown to reduce the viability and proliferation of both SiHa and C33A cervical cancer cells, whereas LGALS1 overexpression significantly enhanced the viable and proliferative rates of SiHa cells in the present study. However, although the number of BrdU positive C33A cells was significantly elevated after upregulation of LGALS1, the viability of C33A cells with LGALS1 overexpression did not change. Moreover, LGALS1 overexpression promoted cervical cancer growth in mice by *in vivo* study. Interestingly, one group has previously indicated that LGALS1 inhibited cell proliferation and induced apoptosis in human colorectal 28 and prostate cancer cells 29. Moreover, decrease of LGALS1 expression has been demonstrated to suppress the proliferation of A172 and U343 but had no effect on U118 and U87 glioma cells 30, 31. Hence, LGALS1 may exhibit dual functions on proliferation in a tissue and cellular context dependent manner in different human malignant tumors or cell lines.

Numerous signaling molecules have been reported to be involved in cell apoptotic pathways, of which the Bcl-2 and Bax family proteins are the best characterized 32. It is widely accepted that Bcl-2 protein-mediated apoptosis blockade contributes to malignant transformation in multiple human cancers 33. Conversely, Bax protein enhances the permeability of the mitochondrial outer membrane, induces release of cytochrome c and consequently promotes apoptosis, which can be counteracted by Bcl-2 33. In this study, overexpression of LGALS1 inhibited the apoptosis of both SiHa and C33A cells while opposite results were obtained after LGALS1 downregulation. Furthermore, LGALS1 knockdown resulted in a sharp decline in the Bcl-2 protein expression in both SiHa and C33A cell lines accompanied with no variation in the level of Bax protein, suggesting that LGALS1 influenced the apoptosis of cervical cancer cells mainly through regulating the expression of Bcl-2 but not Bax. However, the underlying mechanism involved in LGALS1-mediated regulation of Bcl-2 remains to be explored in the future. Intriguingly, accumulated evidences have revealed that the sensitivity to cisplatin is conferred by suppression of Bcl-2 expression in various types of human cancers 34-37. Indeed, it has been documented that elevated levels of LGALS1 significantly induced cisplatin resistance in ovarian cancer partly via upregulation of Bcl-2 38. Moreover, our previous study using proteomics profiling has also confirmed that expression of LGALS1 was significantly decreased in cervical cancer patients after neoadjuvant chemotherapy administration 15. These data collectively indicate that the sensitivity to chemotherapies may be regulated by LGALS1 expression in cervical cancer patients, which also needs to be further validated.

Cell migration and invasion is a complex process initiating from proteolytic degradation of extracellular matrix (ECM) 39. It is well-known that matrix metalloproteinases (MMPs) have the ability to degrade almost all components of ECM and basement membrane, thereby facilitating the invasion and migration of cancer cells 40. Moreover, augmented levels of MMPs are functionally correlated with cancer cell metastasis 41. MMP-2 and MMP-9 belong to gelatinase-A and gelatinase-B, respectively, and are activated in several human cancers as the key enzymes controlling the rate of cell invasion and metastasis 42, 43. Subsequently, Ezrin and Fascin are also found to play important roles in the tumorigenesis and metastasis of several malignancies. Ezrin is closely associated to p85 subunit, which activates the PI3K/Akt pathway and plays an important role in modulating Ewing's sarcoma cell survival, invasion, and metastasis 44. Fascin-1, a member of cytoskeleton proteins, induces the formation of filopodia, lamellipodia, and microspikes of the cell membrane after cross-linking with F-actin, by facilitating the movement, metastasis, and invasion of tumor cells 45. Multiple investigations have speculated that overexpression of Fascin-1 was associated with a variety of human malignancies, such as pancreatic 46, esophageal 47, breast 48 and colorectal cancers 49. In line with these notions, our study provided further evidence to satisfy an unmet clinical need. Our study also found that overexpression of LGALS1 significantly promoted the migration and invasion of both SiHa and HeLa cells, whereas LGALS1 knockdown led to opposing results. Biochemically, we also observed that LGALS1 overexpression was significantly correlated with higher expression of MMP-2, MMP-9, Fascin, Ezrin protein expression in SiHa and C33A cells, while treating with LGALS1-shRNA markedly decreased the expression of these proteins. These results suggested that the oncogenic effects of LGALS1 on cervical cancer migration and invasion could be regulated by the downregulation of MMP-2, MMP-9, Fascin and Ezrin. Several signaling pathways in which LGALS1 is involved have been shown to promote the progression of cancer cells, such as H-Ras-dependent pathway 50, MAPK JNK/p38 pathway 51, and Akt/ERK1/2 signaling pathway 52. However, more studies should be carried out to verify whether these pathways regulate the invasion and migration through altering the level of MMP-2, MMP-9, Fascin and Erzin proteins.

Up to date, several clinicopathological studies have revealed an independent role of LGALS1 for predicting tumor progression and poor survival of cervical cancer patients 17-19. However, the research focusing on the role of LGALS1 in cervical cancer cells has not been extensively conducted. One previous study by Kim *et al.* 18 first showed that knockdown of LGALS1 using small interfering RNA (siRNA) significantly inhibited the proliferation and invasion of SiHa and HeLa cells, which were further confirmed in the current study. Strikingly, our study used shRNA to downregulate LGALS1 more stably and explored several signaling molecules related to the biological behavior of cervical cancer cells, which strengthened the results of this study. Unfortunately, due to the loss of several relevant information of subjects, the association between LGALS1 expression and these clinicopathological parameters could not be analyzed. Moreover, we did not explore how LGALS1 promoted the tumorigenesis and progression of cervical cancer and the potential signaling pathways, which need to be elucidated in the future. Further studies are also warranted to elucidate whether LGALS1 could regulate cisplatin chemosensitivity of squamous cervical cancer cells and the underlying mechanisms.

Taken together, this study revealed that LGALS1 is overexpressed in cervical cancer patients and cervical cancer cells, while LGALS1 downregulation had the opposed results in cervical cancer cells, indicating that LGALS1 may play an oncogenic role in the cervical carcinogenesis. LGALS1 overexpression in cervical cancer cell lines resulted in the promotion of cell proliferation, migration and invasion, as well as suppression of cell apoptosis. Moreover, LGALS1 overexpression also promoted the tumor growth of cervical cancer *in vivo*. Furthermore, LGALS1 downregulation had the opposed results in cervical cancer cells. Due to that downregulation of LGALS1 might contribute to the interference with tumor progression, LGALS1 could be evolved as a promising therapeutic target in the treatment of cervical cancer.

## Figures and Tables

**Table 1:** Description of the selected genes and specific primers used in this study

Gene	Primer name	Primer sequence (5'-3')
LGALS1	LGALS1 (XhoI). F	AATCTCGAGATGGCTTGTGGTCTGGTCGC
	LGALS1 (BamHI). R	TTGGATTCTCAGTCAAAGGCCACACATT
LGALS1-shA	LGALS1-shRNA-A	GATCCGGGTCGCCAGCAACCTGAATCTTCAAGAGAGATTCAGGTTGCGGCGACCTTTTTTG
LGALS1-shB	LGALS1-shRNA-B	GATCCGACCTGTGCCTGCACTTCAACCTCAAGAGGGTTGAAGTGCAGGCACAGGTTTTTTTG
LGALS1-shC	LGALS1-shRNA-C	GATCCGGCCAGATGGATACGAATTCAATCAAGAGTTGAATTCGTATCCATCTGGCTTTTTTG
LGALS1-shD	LGALS1-shRNA-D	GATCCGGCCATCAACTACATGGCAGCTTCAAGAGAGCTGCCATGTAGTTGATGGCTTTTTTG

**Abbreviations:** LGALS1, galectin-1; shRNA, small hairpin RNA.

## Abbreviations

LGALS1: galectin-1
IHC: immunohistochemistry
DMEM: Dulbecco's Modifed Eagle medium
FBS: fetal bovine serum
EDTA: Ethylene Diamine Tetraacetic Acid
ICC: immunocytochemistry
PBS: phosphate-buffered saline
DAB: phosphate-buffered saline
shRNA: small hairpin RNA
SDS-PAGE: sodium dodecyl sulfate-polyacrylamide gel
PVDF: polyvinylidene difluoride
ECL: enhanced chemiluminescence
CCK-8: Cell Counting Kit-8
SPF: specific pathogen free
ROS: reactive oxygen species
ECM: extracellular matrix
MMPs: matrix metalloproteinases
EMT: epithelial-mesenchymal transition
CSC: cancer stem cell
