# Effects of Galectin-1 on Biological Behavior in Cervical Cancer: Erratum

**DOI:** 10.7150/jca.73685

**Published:** 2022-04-25

**Authors:** Mandika Chetry, Yizuo Song, Chunyu Pan, Ruyi Li, Jianan Zhang, Xueqiong Zhu

**Affiliations:** Department of Obstetrics and Gynecology, the Second Affiliated Hospital of Wenzhou Medical University, Wenzhou, Zhejiang, 325027, China.

Since online publication of this article 1, the authors recently noticed that three inadvertent mistakes in the preparation of the figures due to our carelessness. The authors would like to correct them.

(1) The statistical graph of BrdU (+) cells number for C33A cells after LGALS1 overexpression in Figure 3D was misplaced. The authors had inserted the graph of LGALS1-Lt group for SiHa cells in the same panel by mistake. The right statistical graph has been provided to show the BrdU (+) cells number for C33A cells in Figure 3D.

(2) The flow cytometric image of Control-shRNA group for C33A cells in Figure 4D represented an erroneous duplication of the image of Control-Lt group for SiHa cells in Figure 4A. The authors have put the right picture to show the flow cytometric image for Control-shRNA group of C33A cells in Figure 4D.

(3) The statistical graph showing the relative level of Fascin protein for C33A cells in Figure 6D were misplaced. Due to our carelessness in the revision of the figure, the graphs of Fascin and Ezrin proteins level for C33A cells in Figure 6D were duplicated. The corrected graph for Fascin protein level in Figure 6D has been provided.

The correction does not affect the original results and conclusions of this study. The authors apologize for any inconvenience or misunderstanding caused.

## Figures and Tables

**Figure 3:**
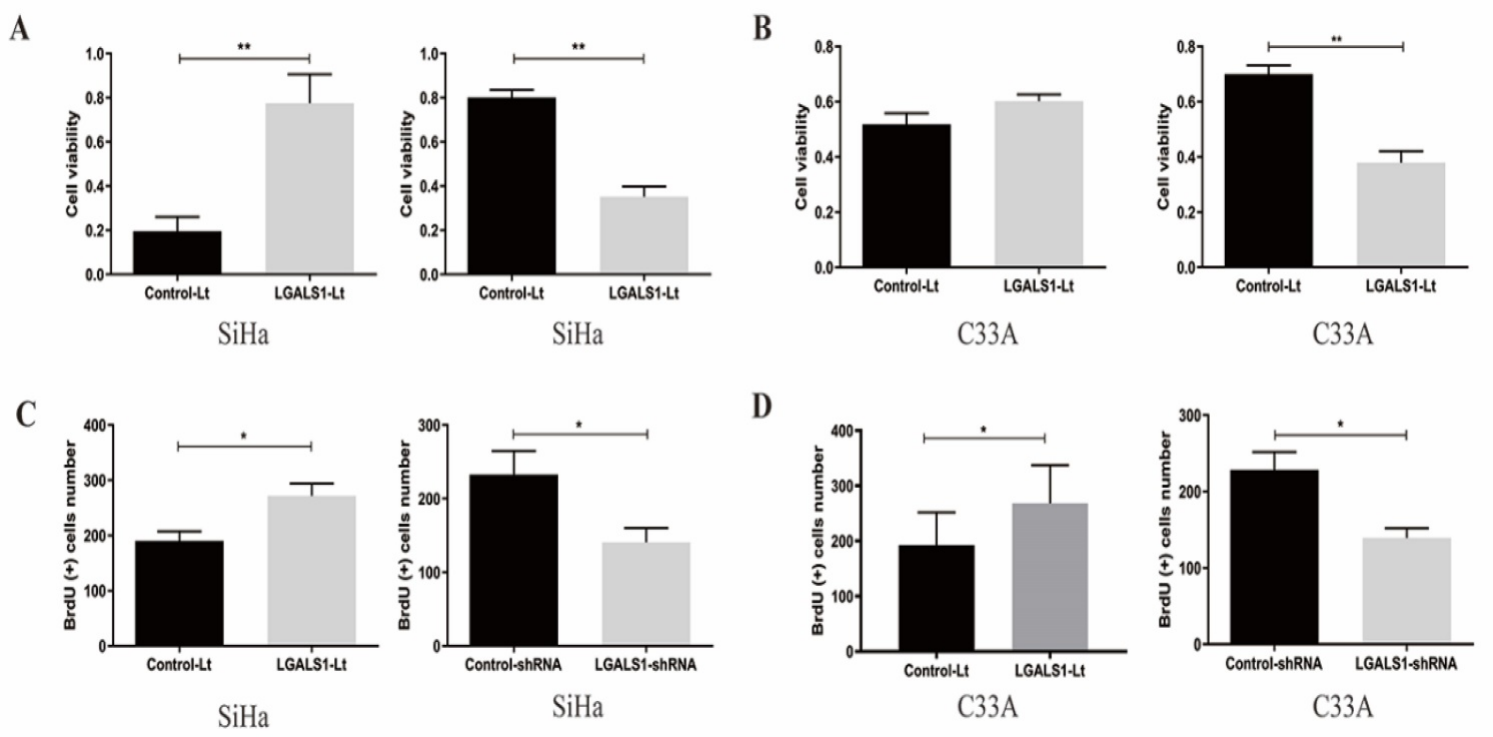
The corrected new figure.

**Figure 4:**
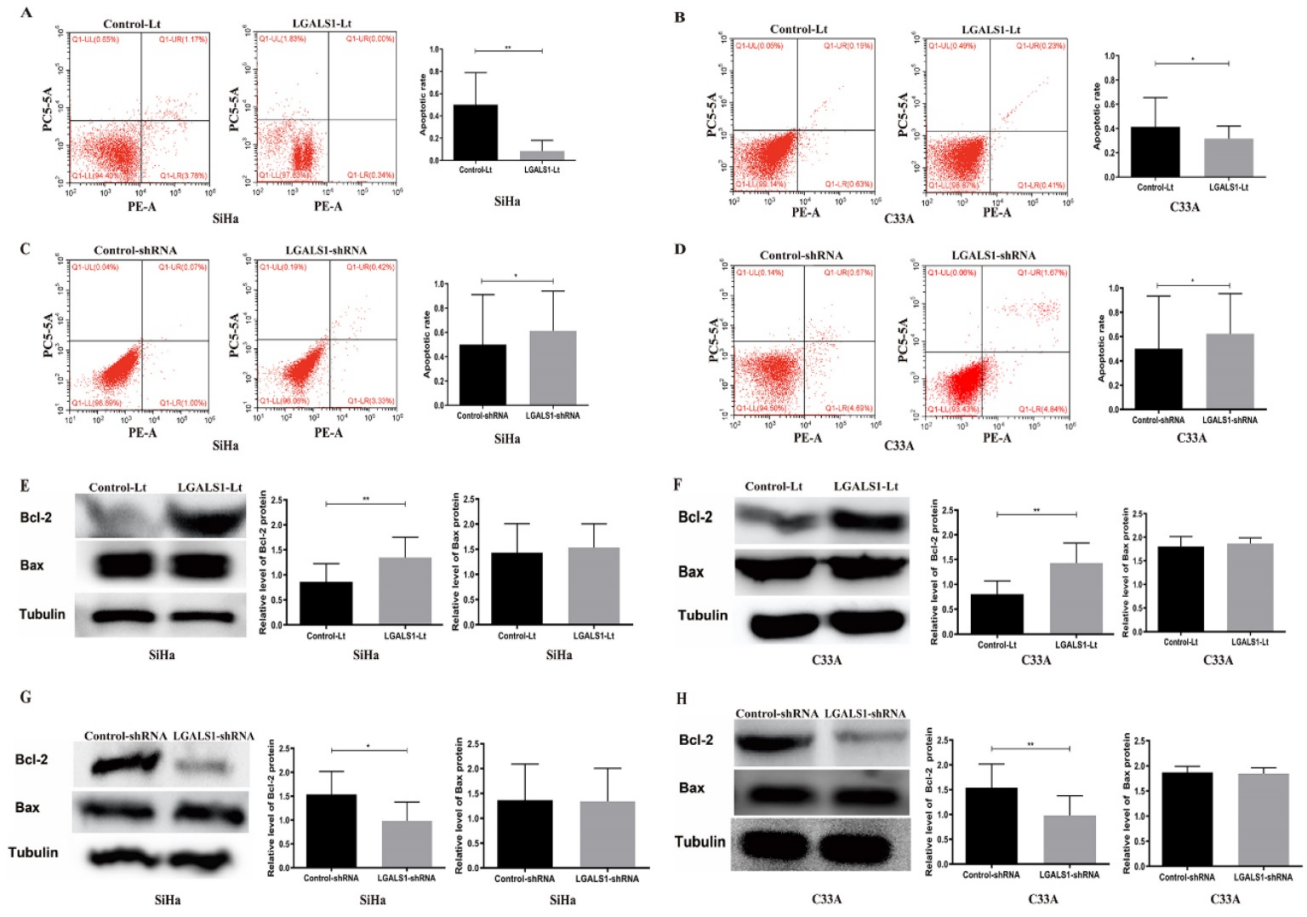
The corrected new figure.

**Figure 6:**
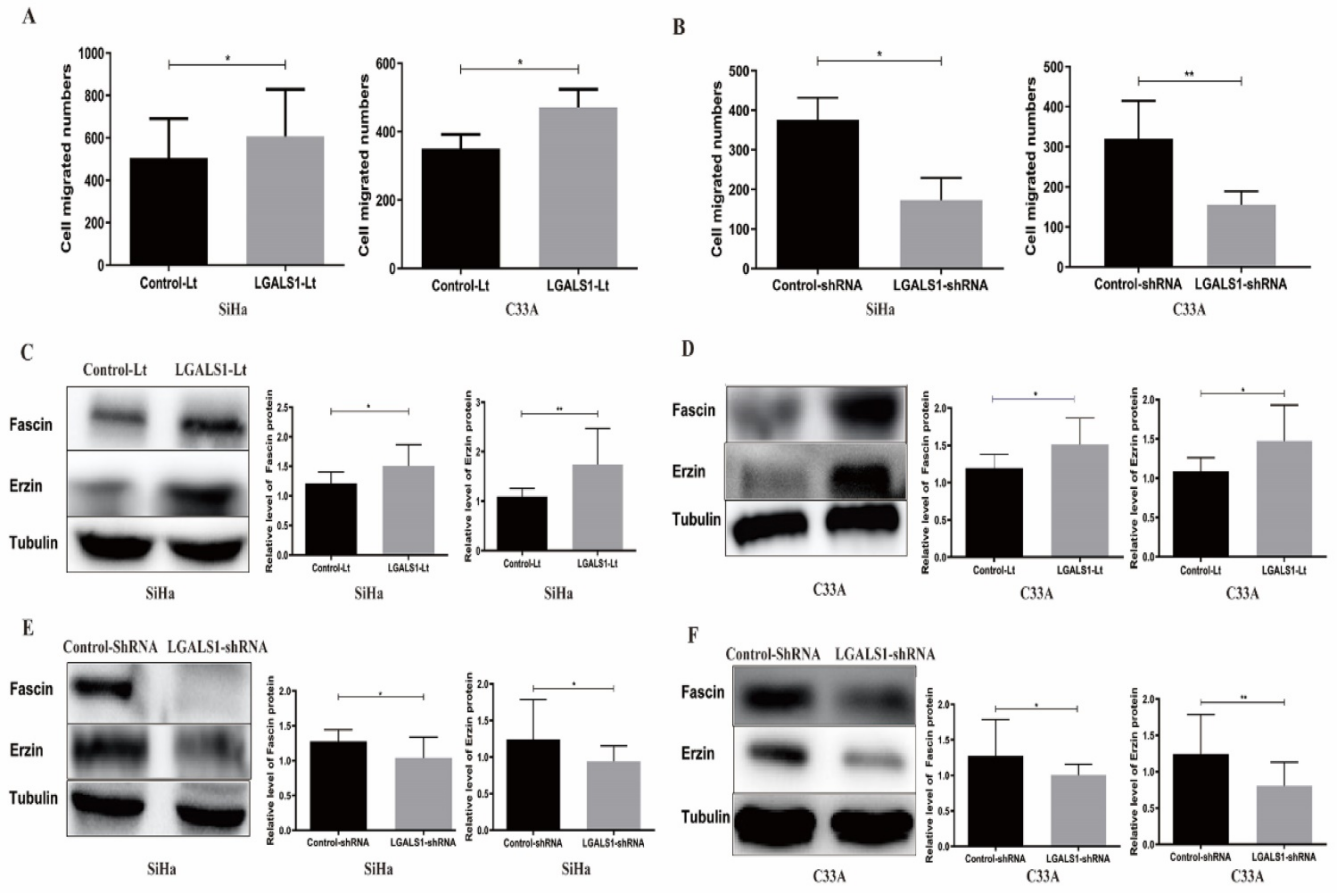
The corrected new figure.
